# Temporal Trends in Cardiovascular Health Status Among Chinese School-Aged Children From 1989 to 2018: Multiwave Cross-Sectional Analysis

**DOI:** 10.2196/45564

**Published:** 2023-10-23

**Authors:** Xijie Wang, Bin Dong, Feifei Huang, Ji Zhang, Rongxin He, Shufa Du, Jiguo Zhang, Jun Ma, Huijun Wang, Bing Zhang, Wannian Liang

**Affiliations:** 1 Vanke School of Public Health Tsinghua University Beijing China; 2 Institute of Child and Adolescent Health School of Public Health Peking University Beijing China; 3 National Institute for Nutrition and Health Chinese Center for Disease Control and Prevention Beijing China; 4 School of Population Medicine and Public Health Chinese Academy of Medical Sciences and Peking Union Medical College Beijing China; 5 Department of Nutrition and Carolina Population Center University of North Carolina at Chapel Hill Chapel Hill, NC United States

**Keywords:** cardiovascular health, school-aged children, temporal change, China Health and Nutrition Survey

## Abstract

**Background:**

Despite the release of updated metrics for Life’s Essential 8 (LE8), key indicators for assessing cardiovascular health (CVH) status, there is currently no report on their distribution among Chinese children.

**Objective:**

This study aimed to assess the nationwide distribution of CVH in Chinese school-aged children using LE8 scores and analyze temporal changes in these scores over time.

**Methods:**

Participants aged 7 to 19 years from 11 waves (between 1989 and 2018) of the China Health and Nutrition Survey were included in this study. LE8 components were grouped into 2 domains of health behaviors (diet, physical activity, nicotine exposure, sleep) and health factors (BMI, blood lipids, blood glucose, blood pressure). Scores of overall CVH and each LE8 metric were calculated individually. Temporal changes were assessed with joint point regression models by rural and urban living residence. The causal relationships between health behaviors and health factors that changed the most over time were built with cross-lagged panel models.

**Results:**

A total of 21,921 participants, 52.6% (n=11,537) of whom were male, who had data for at least 4 CVH components were included in the analysis. The mean age was 13 (SD 3.6) years. The overall CVH score remained stable in most regions, with the lowest found in Shandong from East China, which had a mean between 67 (SD 10.9) and 67.2 (SD 12.4). In contrast, the highest score was found in Guizhou from Southwest China, with a mean between 71.4 (SD 10.8) and 74.3 (SD 10.3). In rural areas, the diet score decreased significantly from 1997 onward with a speed of 0.18 (95% CI: 0.15-0.21; *P*<.001) per year, and the BMI score decreased significantly from 2005 onward with a speed of 0.56 (95% CI 0.44-0.68; *P*<.001) per year. In urban areas, the diet score decreased from 1994 onward with a speed of 0.03 (95% CI: 0.001-0.07; *P*=.04) per year, and the BMI score decreased from 2002 onward with a speed of 0.63 (95% CI 0.47-0.79; *P*<.001) per year. The sleep score dropped constantly in both urban and rural areas, with a speed of 0.69 (95% CI 0.58-0.80; *P*<.001) and 0.69 (95% CI: 0.52-0.86; *P*<.001) per year, respectively. A decline in the diet score led to a decline in the BMI score with a coefficient of 0.190 (95% CI 0.030-0.351; *P*=.02), while a decline in the BMI score led to a decline in sleep health with a coefficient of 0.089 (95% CI 0.010-0.168; *P*=.03).

**Conclusions:**

Chinese school-aged children and adolescents were generally of moderate CVH status, but mutual influences existed between CVH metrics. Dietary interventions should be prioritized for promoting overall CVH in the future.

## Introduction

Cardiovascular disease (CVD) is the leading cause of death and disability in the world, in large part because of risk factors modifiable by changes in behavior [1,2]. Individuals with an unfavorable cardiovascular health (CVH) status during childhood are more likely to develop subclinical atherosclerosis and subsequent CVD later in life [3]. Controlling and reducing CVD-related mortality and morbidity are not only one of the key indicators of the United Nations’ Sustainable Development Goals [2] but also one of the top priorities of the 2030 Healthy China Plan [4].

Although the overall CVD mortality has stalled in recent years, especially in middle- and low-income countries [5], efficient prevention strategies could further effectively reduce age-adjusted death rates due to CVD [6,7]. This points to the importance of recognizing, evaluating, and maintaining overall CVH.

The newly released Life’s Essential 8 (LE8), key measures for improving heart health released by the American Heart Association, provide a comprehensive tool to evaluate overall CVH status and specific metrics of CVH behaviors or factors. Compared with the previous version, Life’s Simple 7 [8], the new version provides not only classifications of CVH status but also continuous scores that are helpful for both cross-sectional and longitudinal analyses on differences and trends in CVH status distribution. The revised version incorporates newly identified risk factors, such as sleep health, alongside metrics that have demonstrated stronger associations with long-term CVD, such as the replacement of total cholesterol with non–high-density lipoprotein (HDL) cholesterol [9,10].

The newly released metrics have garnered significant attention in the field of CVH research. However, prior studies have not addressed either the national distribution of CVH status among Chinese school-aged children or the temporal changes in CVH metrics over time. Accordingly, in this study, we collected data pertaining to children and adolescents aged 7 to 19 years from waves spanning 1989 to 2018 in the China Health and Nutrition Survey (CHNS). We aimed to analyze temporal trends and geographical variations in CVH in Chinese school-aged children. Additionally, we aimed to investigate whether changes in health behaviors over the past 3 decades have had an impact on health factors.

## Methods

### Study Participants

The study data were obtained from 11 waves of the CHNS, an open prospective cohort study carried out by the University of North Carolina at Chapel Hill in partnership with the National Institute for Nutrition and Health of the Chinese Center for Disease Control and Prevention [11]. The baseline survey was conducted in 1989 and followed up in 1991, 1993, 1997, 2000, 2004, 2006, 2009, 2011, 2015, and 2018, respectively.

This project covered 15 provinces (autonomous regions and municipalities) including Heilongjiang, Liaoning (did not participate in 1997), Jiangsu, Shandong, Henan, Hubei, Hunan, Guangxi, Guizhou, Beijing, Shanghai, Chongqing, Shaanxi, Zhejiang, and Yunnan. Notably, Heilongjiang only participated from 1997 onward; Beijing, Chongqing, and Shanghai participated from 2011 onward; and Zhejiang, Yunnan, and Shaanxi participated from 2015 onward. A multistage, stratified, clustered random method was used for sampling. Based on the national administrative regions, a weighted sampling scheme was used to randomly select 2 cities and 4 counties from each province. Next, 2 urban and 2 suburban neighborhoods were randomly selected in each city, and 1 township and 3 villages were randomly selected in each county. Urban neighborhoods in the cities and townships in the counties were grouped into urban areas, and suburban neighborhoods in the cities and villages in the counties were grouped into rural areas. More design details are described elsewhere [11,12].

### Study Measurements

#### Dietary Survey

A 3-day consecutive 24-hour-dietary recall method was employed to gather information on the consumption of all foods and drinks. Due to notable variations in the Chinese diet between weekdays and weekends, we recorded the consumption for 2 workdays and 1 weekend day. After training, investigators conducted face-to-face interviews, asking participants about their food and beverage intake over the past 24 hours for 3 consecutive days. Simultaneously, a household weighing method was used to document the consumption of cooking oil and condiments over the corresponding 3 days. The allocation of cooking oil and condiment consumption to individuals was based on how often they cooked at home, meal proportions, and the ratio of individual energy intake to household energy intake. To minimize the recall bias, respondents were asked to write down what they ate and drank every day.

Food items were categorized according to the categories outlined in the China Food Composition Tables. In this study, the term “milk” referred to the equivalent of liquid milk, encompassing various dairy and dairy products converted based on the protein content of every 100 grams of the edible portion. The category “beans” included soybeans, other legumes, and their products. The intakes of soybean flour and soybean milk were converted into the equivalent of soybeans, considering the protein content of every 100 grams of the edible portion. Salt intake consisted of sodium chloride (NaCl) from cooking salt, paste, and sauce.

#### Anthropometric Measurements

The participants’ height, weight, systolic blood pressure (SBP), and diastolic blood pressure (DBP) were measured by trained investigators. Height was recorded to the nearest 0.1 centimeters. Weight was recorded to the nearest 0.1 kilograms. BMI was calculated as weight (kg)/height^2^ (m^2^).

#### Blood Sample Collection

In the years 2009, 2015, and 2018, the CHNS project collected fasting venous blood samples from participants aged 7 years and above. The fasting plasma glucose (FPG) concentration was measured using the GOD-PAP (glucose oxidase-phenol-4-aminoantipyrineenzymatic) method (Randox Laboratories Ltd). Glycated hemoglobin (HbA1c) levels were measured using a high-performance liquid chromatography system (model HLC-723 G7; Tosoh Corp). Participants’ diagnosis of diabetes mellitus was confirmed based on doctor diagnoses, as reported by participants themselves or by their parents in cases where adolescents could not answer. Total cholesterol and HDL cholesterol were measured using the CHOD-PAP (cholesterol oxidase: P-aminophenazone) method (Kyowa Medex Co Ltd). Non-HDL cholesterol was calculated as the difference between total cholesterol and HDL cholesterol.

#### Nicotine Exposure

Nicotine exposure was assessed through active smoking and secondhand smoke exposure. Active smoking was categorized into three groups: (1) individuals who had never smoked, (2) those who had smoked at any point or had quit smoking for more than 30 days, and (3) those who were currently smoking or had quit smoking within the last 30 days. Secondhand smoke exposure was defined as living with people who smoke.

#### Physical Activity

Physical activity data, comprehensively collected in this project since 2000, were obtained through questionnaires. Physical activity encompassed occupational, domestic, travel, and leisure activities.

#### Sleep Duration Data

Sleep duration data, including both night sleep time and daytime sleep time, were collected from questionnaires as part of the study.

### Quantification of CVH Components

The methods for measuring, evaluating, and quantifying the 8 CVH components are displayed in Tables S1 and S2 of Multimedia Appendix 1. These methods closely aligned with the original LE8 metrics tailored for children and adolescents under the age of 19 years. The 8 components comprised of 4 health behaviors (diet, physical activity, nicotine exposure, and sleep health) and 4 health factors (BMI, non-HDL cholesterol, FPG, and blood pressure), as defined by the LE8 reference. Furthermore, sleep health was evaluated in accordance with the Healthy China Initiative (2019-2030), which recommends a sleep duration of 10 hours for primary school students, 9 hours for secondary school students, and 8 hours for high school students. Age and sex-specific BMI percentiles were generated based on growth references from the World Health Organization [13]. SBP and DBP were transformed into age, sex, and height–specific percentiles, and evaluated based on the American Academy of Pediatrics’ Clinical Practice Guideline for Screening and Management of High Blood Pressure in Children and Adolescents, released in 2017 [14].

### Statistical Analyses

Each of the LE8 components was scored on a scale ranging from 0 to 100, with the total CVH score calculated as the average of the 8 component scores. Overall CVH scores between 80 and 100 were categorized as high CVH, scores between 50 and 79 were categorized as moderate CVH, and scores between 0 and 49 were classified as low CVH. The mean and SD were calculated for continuous variables, and numbers and percentages were calculated for categorical variables. The median and IQR were reported for non-HDL cholesterol. Geographical regions were categorized as North China, Northeast China, East China, Central China, Southwest China, and Northwest China according to codes for the administrative divisions of the People’s Republic of China (GB/*t* 2260), as outlined in Table S3 of Multimedia Appendix 1. Joint point regression models were used to analyze the differences in CVH scores over time, considering rural or urban living residence. Adjustments were made for the children’s age and sex as appropriate. Since scores for both health behaviors and health factors decreased sharply after 2004, cross-lagged panel models were applied to explore causal relationships between changes in health behaviors and health factors. Model fit was evaluated based on the Comparative Fit Index and Tucker-Lewis Index scores greater than 0.95, with a root mean squared error of approximation less than 0.05 being indicative of good fit [15]. All statistical analyses were performed using Stata software (version 14.0; StataCorp). The associations were considered significant at 2-sided *P*<.05.

### Ethics Approval

This study was approved by the institutional review board of the University of North Carolina at Chapel Hill and the National Institute for Nutrition and Health of the Chinese Center for Disease Control and Prevention (2018-004). Participants aged 7 to 17 years provided signed informed consent from themselves and their guardians, while participants aged 18 years and above provided their own signed informed consent forms to participate in the original surveys. The data for our study were completely deidentified and did not contain any information that could be traced back to the individual participants.

## Results

In total, 21,921 (77%) out of 28,477 participants, 52.6% (n=11,537) of them male, aged between 7 and 19 (mean 13, SD 3.6) years, with data for at least 4 CVH components, were included in the study analysis. Among the participants, 6071 (27.7%) were from urban areas. Demographic characteristics, as well as CVH status, are detailed in Table 1 by geographic regions, and comparisons of demographic characteristics between the excluded and included children are displayed in Table S4 of Multimedia Appendix 1. The sample distribution at each research wave is displayed in Table S5 of Multimedia Appendix 1.

**Table 1:** Demographic characteristics of participants by geographic regions.

Characteristics		Overall	North China (n=403)	Northeast China (n=2746)	East China (n=4271)	Central China (n=10,208)	Southwest China (n=3830)	Northwest China (n=463)	Difference (*P* value)
Urban area, n (%)		6071 (27.7)	321 (79.7)	643 (23.4)	1418 (33.2)	2561 (25.1)	992 (25.9)	136 (29.4)	<.001
Male sex, n (%)		11,537 (52.6)	213 (52.9)	1418 (51.6)	2242 (52.5)	5420 (53.1)	2005 (52.4)	239 (51.6)	.803
Age (years), mean (SD)		13 (3.6)	12.6 (3.5)	13.1 (3.5)	13.1 (3.6)	13 (3.6)	12.9 (3.6)	12.2 (3.2)	<.001
Diet score, mean (SD)		28.4 (9)	28.8 (10.8)	30.1 (10.4)	28.9 (9.4)	28.2 (8.7)	27.2 (7.7)	26.7 (7.4)	<.001
**Physical activity, n (%)**									<.001
	≥420 min	5477 (30.8)	163 (40.7)	615 (26.9)	907 (26.7)	2343 (29.2)	1292 (40.3)	157 (34.7)	
	360 to 419 min	701 (4)	15 (3.7)	86 (3.8)	132 (3.9)	318 (4)	133 (4.2)	17 (3.8)	
	300 to 359 min	940 (5.3)	19 (4.7)	142 (6.2)	156 (4.6)	395 (4.9)	207 (6.5)	21 (4.6)	
	240 to 299 min	1041 (5.9)	25 (6.2)	150 (6.6)	182 (5.4)	443 (5.5)	219 (6.8)	22 (4.9)	
	120 to 239 min	3259 (18.3)	73 (18.2)	499 (21.8)	534 (15.7)	1482 (18.5)	596 (18.6)	75 (16.6)	
	1 to 119 min	3300 (18.6)	55 (13.7)	446 (19.5)	728 (21.5)	1575 (19.6)	413 (12.9)	83 (18.3)	
	Never	3049 (17.2)	51 (12.7)	350 (15.3)	755 (22.3)	1469 (18.3)	346 (10.8)	78 (17.2)	
**Cigarette use, n (%)**									<.001
	Never tried	10356 (94.8)	205 (99.5)	1348 (94.4)	2203 (98.2)	4629 (94.4)	1763 (91.4)	208 (99.5)	
	Tried but >30 days ago	13 (0.1)	—^a^	3 (0.2)	1 (0)	5 (0.1)	4 (0.2)	—	
	Used within 30 days	552 (5.1)	1 (0.5)	77 (5.4)	39 (1.7)	271 (5.5)	163 (8.5)	1 (0.5)	
Sleep per night (hours), mean (SD)		8.7 (1.2)	8.4 (1.1)	8.5 (1.2)	8.6 (1.2)	8.8 (1.2)	8.9 (1.1)	8.4 (1.1)	<.001
Height (cm), mean (SD)		146.6 (16.8)	153 (17.6)	150.9 (16.5)	149.8 (16.7)	145 (16.5)	143.1 (16.3)	149.5 (16.2)	<.001
Weight (kg), mean (SD)		40.1 (14.4)	48.8 (17.1)	43.5 (14.6)	43.8 (14.7)	38.1 (13.2)	37.6 (15.1)	42.8 (14.1)	<.001
BMI (kg/m^2^), mean (SD)		18 (3.2)	20.2 (4.3)	18.6 (3.4)	19 (3.4)	17.5 (3)	17.7 (3)	18.7 (3.6)	<.001
Non-HDL^b^ cholesterol (mg/dL), median (IQR)		94.9 (33.3)	104.6 (45.8)	96.2 (30.9)	99.8 (38.3)	95.5 (32.5)	94 (30.9)	84.3 (32.1)	<.001
Fasting plasma glucose (mg/dL), mean (SD)		89.9 (13.9)	91.2 (10.5)	86.9 (17.1)	88.6 (9.9)	91.3 (17.5)	90 (11.5)	90.1 (8.4)	<.001
Systolic blood pressure (mmHg), mean (SD)		99.9 (13.5)	106.4 (11.7)	102.6 (13.9)	102.6 (13.7)	98.9 (13)	96.8 (13.1)	100.4 (13.5)	<.001
Diastolic blood pressure (mmHg), mean (SD)		65.4 (9.8)	68.5 (7.6)	68 (10.4)	66.6 (9.8)	64.7 (9.5)	63.7 (9.6)	66.7 (8.7)	<.001

**Figure 1:**
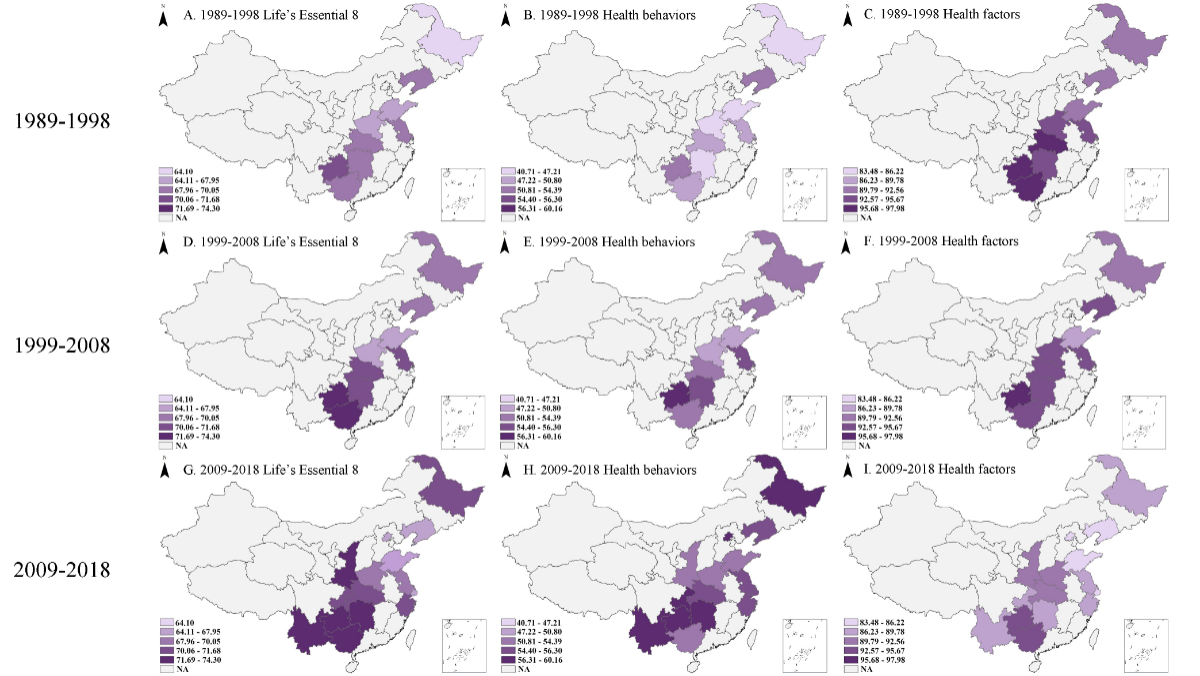
Distribution and temporal change of overall cardiovascular health (CVH) status in Chinese school-aged children and adolescents from 1989 to 2018 based on natural breaks (Jenks) classification. A. Life’s Essential 8 score (1989-1998); B. Health behavior score (1989-1998); C. Health factor score (1989-1998); D. Life’s Essential 8 score (1999-2008); E. Health behavior score (1999-2008); F. Health factor score (1999-2008); G. Life’s Essential 8 score (2009-2018); H. Health behavior score (2009-2018); I. Health factor score (2009-2018).

During the past 3 decades, the overall CVH score remained stable in most of the involved provinces, with the lowest score observed in Shandong from East China, with a mean between 67 (SD 10.9) and 67.2 (SD 12.4). The highest score was observed in Guizhou from Southwest China, with a mean between 71.4 (SD 10.8) and 74.3 (SD 10.3). The most significant change in overall CVH status was observed in Heilongjiang from Northeast China, with scores increasing from a mean of 64.1 (SD 11.1) to 71.1 (SD 10.3). Health behavior scores, though much lower than overall scores, showed a gradual increase during the decades. The greatest increase was found in Guizhou from Southwest China and Hunan from Central China, with health behavior scores increasing from a mean of 50.8 (SD 18.1) to 60.2 (SD 14.3) and from 46.8 (SD 17.5) to 56.7 (SD 15.7), respectively. Conversely, health factor scores decreased across all areas, with the biggest decline found in Liaoning from Northeast China (mean 92.3, SD 13.8 to mean 84, SD 17.8) and Shandong from East China (mean 91.6, SD 14.5 to mean 83.6, SD 16.7). Temporal changes in CVH scores by province are shown in Figure 1.

**Figure 2:**
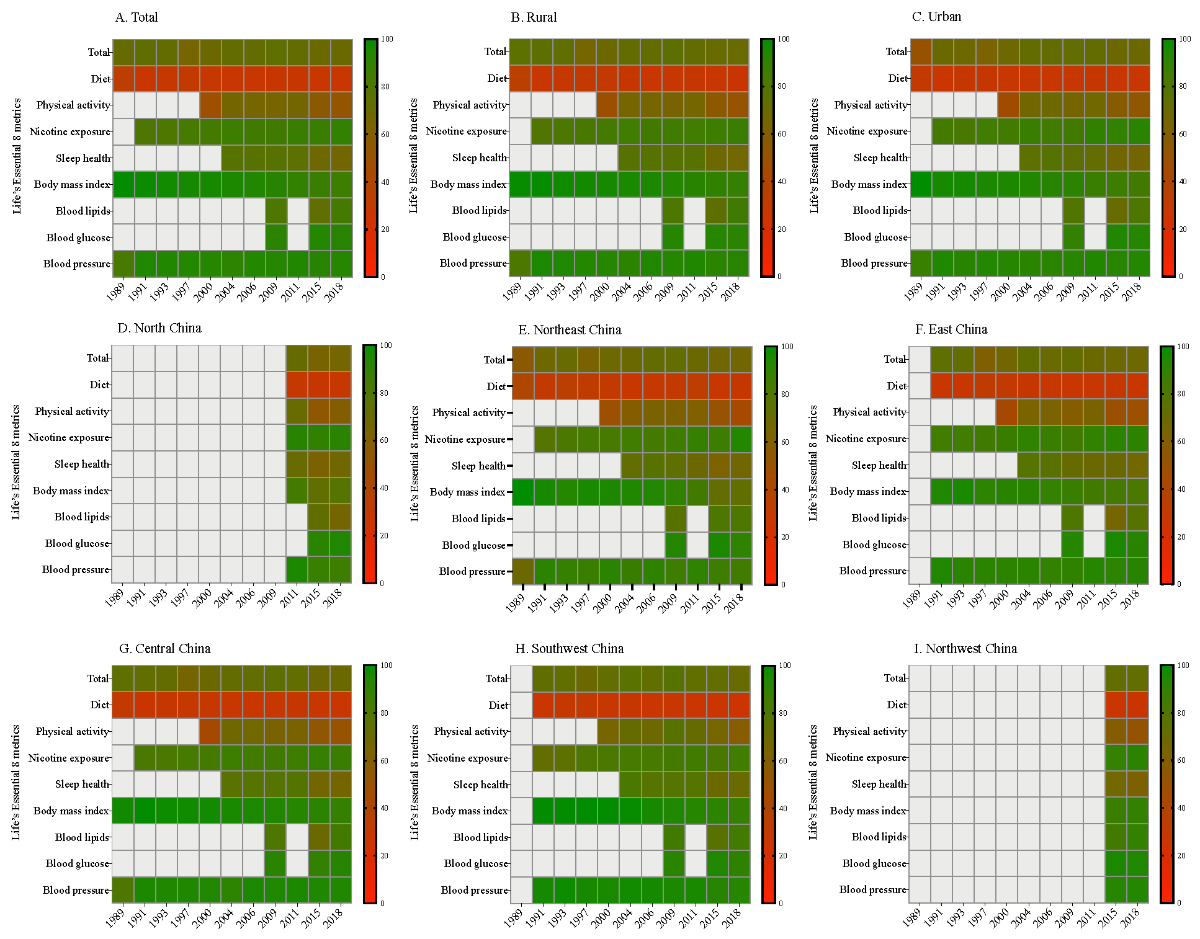
Changes in cardiovascular health metrics for Chinese school-aged children and adolescents from 1989 to 2018 by geographic region.

Regarding specific CVH metrics, the healthy diet score consistently remained the lowest for all research waves and in all 6 geographical regions. Worsened sleep health was observed in all geographical regions, while worsened BMI was particularly prominent in North and Northeast China (Figure 2 and Figure S1 in Multimedia Appendix 1).

**Figure 3:**
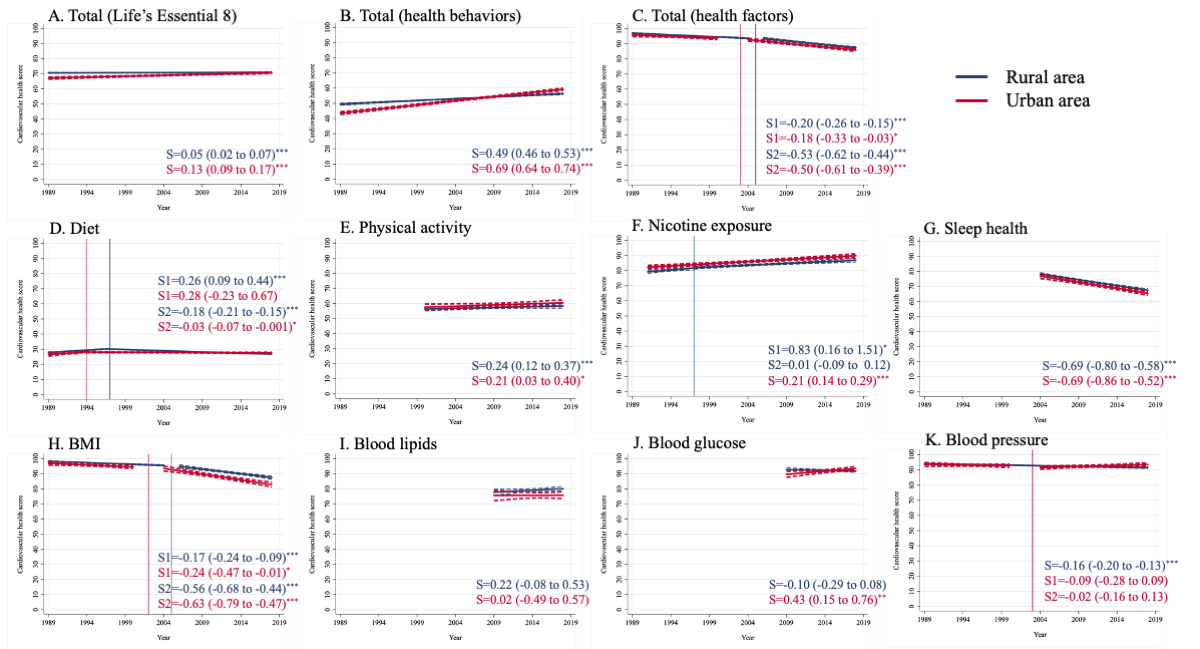
Joint point regression analysis of time trends in cardiovascular health scores in Chinese school-aged children and adolescents from 1989 to 2018. Where a joint point was recognized, S1 referred to the slope before the joint point, and S2 referred to the slope after the joint point. S: slope.

Joint point regression models were then applied to analyze the temporal changes in CVH metrics. Although similar trends were observed in both rural and urban areas, time lags in break points were identified for several metrics (Figure 3). Generally, overall CVH scores and health behavior scores displayed linear growth trends in both rural and urban areas. Total health factor scores decreased faster in rural areas since 2005, with a speed of 0.53 (95% CI 0.44-0.62; *P*<.001) per year, and in urban areas since 2003, with a speed of 0.50 (95% CI 0.39-0.61; *P*<.001). Decreases in diet scores became more significant in rural areas since 1997, with a speed of 0.18 (95% CI 0.15-0.21; *P*<.001) and in urban areas since 1994, with a speed of 0.03 (95% CI 0.001-0.07; *P*=.04). The decrease in BMI scores became more pronounced in rural areas since 2005, with a speed of 0.56 (95% CI 0.44-0.68; *P*<.001) and in urban areas since 2002, with a speed of 0.63 (95% CI 0.47-0.79; *P*<.001). Constant decreases were observed in sleep health scores, with a speed of 0.69 (95% CI 0.58-0.80; *P*<.001) in rural areas and 0.69 (95% CI 0.52-0.86; *P*<.001) in urban areas.

**Figure 4:**
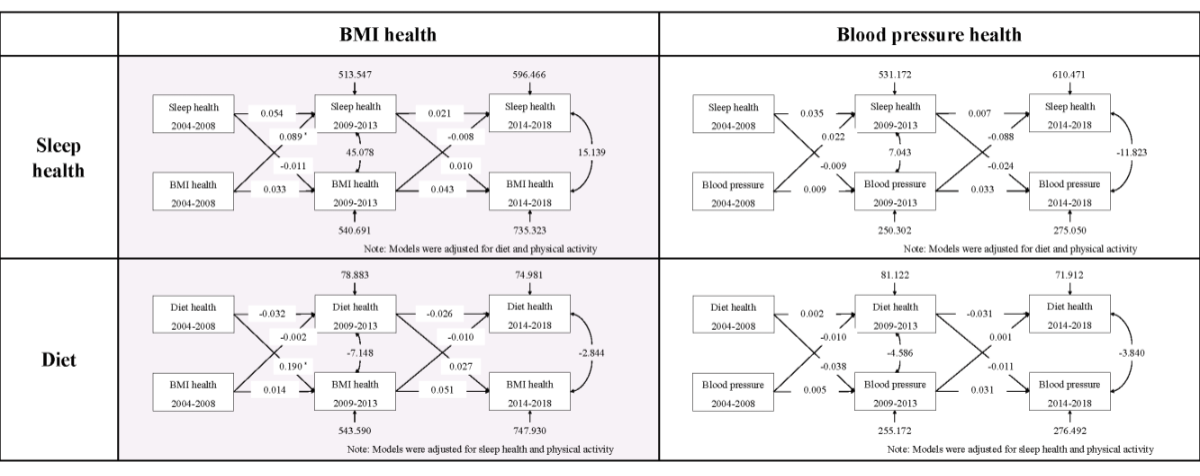
Cross-lagged standard regression coefficient of sleep health, diet health, BMI, and blood pressure health between 2009 and 2013 and 2014 and 2018. BP: blood pressure.*Indicated to *P*<.05.

Cross-lagged panel models were constructed between health behaviors (including diet, physical activity, and sleep health) and health factors (including BMI and blood pressure health) during the study waves spanning 2004 to 2008, 2009 to 2013, and 2014 to 2018 (Figure 4). The results indicated that lowered BMI health between 2004 and 2008 resulted in lowered sleep health between 2009 and 2013, with a coefficient of 0.089 (95% CI 0.010-0.168; *P*=.03). Concurrently, lowered diet scores from 2004 to 2008 resulted in lowered BMI health from 2009 to 2013, with a coefficient of 0.190 (95% CI 0.030-0.351; *P*=.02).

## Discussion

### Principal Findings

To our knowledge, this is the first study examining CVH status and its temporal changes over the past decades in Chinese school-aged children and adolescents using the new LE8 metrics. From 1989 to 2018, the overall LE8 score remained relatively stable at a moderate level. Simultaneously, the overall score for health behaviors moderately increased, while that of health factors declined significantly over time. Therefore, we inferred that Chinese children and adolescents may face a prolonged downward trend in overall CVH status in the future.

The overall level of CVH scores in Chinese children in the recent decade was compatible with those of children in the United States, ranging between 64.6 and 74.1, depending on the metrics used for calculation [16]. Both groups shared suboptimal performance in diet, but Chinese children and adolescents had relatively lower scores in health behaviors such as physical activity and sleep health. The initiation of CVD and related diseases in childhood underscores the critical importance of LE8 metrics, turning childhood and youth into new frontiers for CVD prevention [17]. As the behavioral change theory has emphasized, changes in human behavior should be based on education, enhanced self-motivation, and adequate external support, eventually resulting in changes in health outcomes [18,19]. Early and sustained multicomponent educational programs that focus on health promotion in children and adolescents could represent a critical window of opportunity to potentially prevent disease in later life [20].

The results of our analysis indicated that the overall CVH level was lower in the eastern part of China compared to the western part, similar to findings reported for Chinese adults from the 2021 Report on Cardiovascular Health and Disease in China [21,22]. In contrast with reports from countries in North America, where lower CVH status is typically associated with less favorable economic conditions [23], we found poorer CVH status in areas with better economic conditions in China. This may be attributed to wider socioeconomic gaps and the prevalence of unhealthy lifestyles in urban and higher-income areas [24]. However, further research is needed for a more detailed exploration of the social determinants of CVH.

Several metrics in the LE8 were particularly noteworthy in this study, highlighting persistently low diet quality, fluctuating levels of physical activity, and persistent declines in sleep and BMI health. Of more concern is that although there was a time lag between urban and rural areas, the trend and speed of the simultaneous decline in rural areas for these indicators were comparable—or even greater than—that of urban areas.

Enhancing physical fitness in school-aged children and adolescents has been a top priority for the Chinese government. The State Council of the People’s Republic of China’s initiatives “Suggestions on Strengthening Youth Sports and Enhancing Youth Physical Fitness,” released in 2007 [25], and the “Sunshine Sports Program,” which encourages all school-aged children and adolescents to have at least 1 hour of outdoor physical activity, have been implemented. Nationwide survey findings from both our analysis and the ﻿Chinese National Survey on Students’ Constitution and Health showed a significant improvement in weekly exercise hours and physical fitness during the following years [26,27]. However, our analysis reveals that physical activity scores have continued to decline since 2011, primarily due to lower scores in Northwest China. The geographical imbalance in policy implementation is evident and should be addressed in future efforts.

Sleep health has drawn attention in some high-income countries, and public health initiatives have been implemented to promote better sleep health [28,29]. In the United States, national surveys indicate an improvement in sleep duration due to earlier bedtimes and longer sleep durations in various segments of the population [30]. Insufficient sleep can directly impact growth and development in children and adolescents, acting as a medium to spread the adverse effects of modern social development on CVH through its impact on the endocrine system [31-34].

A population-based analysis found a causal cycle between poor diet, worsened BMI, and shortened sleep time, as all 3 metrics have experienced significant decreases during the latest decade. This suggests that overall CVH in Chinese children and adolescents could soon worsen. The LE8 guideline emphasizes that overall CVH status depends not on a single metric but on a combination of all 8 metrics. Emphasizing certain aspects when policymaking could lead to joint improvements in other metrics, making it crucial to avoid geographical biases in policy implementations.

Our study results indicate that it might be cost-effective and quicker to prioritize diet health interventions for CVH promotion, as diet could impact other CVH metrics both directly and indirectly. Moreover, comprehensive intervention strategies tailored to specific regional conditions are needed for further CVH promotion, emphasizing the need for developing evidence-based behavior change techniques [35].

It is important to note that our study only reflects the temporal change and distribution of CVH status in school-aged children and adolescents until 2018. Since then, a series of government policies, including the “Healthy China Initiative (2019-2030),” “Implementation Plan for Obesity Prevention and Control in Children and Adolescents,” and “Implementation Plan for Comprehensive Prevention and Control of Myopia in Children and Adolescents,” have been released to guide health promotion programs targeting Chinese children and adolescents. Key factors such as the prevalence of obesity and myopia were listed as part of assessment indicators to ensure that local governments attach enough importance to them. Dietary health was also addressed in nearly all the aforementioned policies, with related health education packages to aid in implementation. With such a strong driving force, we anticipate significant improvements in CVH and overall health status for Chinese school-aged children and adolescents by 2030, setting a valuable example for other countries.

### Limitations

This study has several limitations. First, although the study data originated from one of China’s largest and longest-standing nationwide studies of child and adolescent health status, there is an inherent bias in sample selection. Approximately 23% (n=6556) of the participants from the original data set were excluded due to missing data (mainly from blood samples or surveys before 2000). Although the excluded participants were not likely to have influenced the findings of this study, which focuses on CVH changes in the past 20 years, we are unable to ascertain if they would influence the overall CVH scores and their trends before 2000. Nevertheless, the original CHNS sample was not designed to be representative of China but rather to capture a diverse range of economic and demographic circumstances [12] Therefore, caution should be taken when generalizing these findings. Despite these limitations, based on the setting of the original survey, our study results still provide valuable insights into the changing trends of CVH status among children and adolescents from different socioeconomic development levels in China.

Second, although the original CHNS was designed to be a prospective cohort study, our study adopted a multiwave cross-sectional study design due to the lack of personal information. Therefore, the analyses were conducted at the population level, and further studies with individual-level data could provide confirmation of the reported population-level data. Third, concerns about the reliability of the self-reported dietary data have been raised in the scientific community, and we observed lower reported energy intakes in many Chinese children in our previous studies. However, given the complexity of the Chinese diet, objective dietary survey methods are still being developed.

### Conclusions

Based on the latest LE8 metrics, Chinese school-aged children and adolescents generally exhibited a moderate level of CVH over the past decades. Poor performance in health behaviors, including diet, sleep, and physical activity may contribute to worsening overall CVH in the future. Therefore, policies and interventions addressing diet health may be the breakthrough point for promoting overall CVH status in Chinese children and adolescents.

## Appendix

Multimedia Appendix 1 Supplementary figures and tables.

## Abbreviations

CHNS: China Health and Nutrition Survey
CHOD-PAP: cholesterol oxidase: P-aminophenazone
CVD: cardiovascular disease
CVH: cardiovascular health
DBP: diastolic blood pressure
FPG: fasting plasma glucose
GOD-PAP: glucose oxidase-phenol-4-aminoantipyrineenzymatic
HbA1c: glycated hemoglobin
HDL: high-density lipoprotein
LE8: Life’s Essential 8
NaCl: sodium chloride
SBP: systolic blood pressure

## Data Availability

Partial data from 1989 to 2011 are available upon request to the corresponding author. The remaining data are not publicly available.
