# Supplementary material for: Temporal Trends in Cardiovascular Health Status Among Chinese School-Aged Children From 1989 to 2018: Multiwave Cross-Sectional Analysis
Source: JMIR Public Health Surveill. 2023 Oct 23;9:e45564. doi: 10.2196/45564 (PMC10628687; doi:10.2196/45564)
Supplement: Multimedia Appendix 1 [file publichealth_v9i1e45564_app1.docx]

| Table S1. Measurements and quantitative assessment criteria of Life's Essential 8 metrics (health behaviors). | | | | |
| --- | --- | --- | --- | --- |
| CVH metric | | Method of measurement | Tools for evaluation | Quantification |
| Diet | | Consecutive 3 days 24 hours (including 2 workdays and 1 weekday) dietary recall | Mediterranean Eating Pattern for Americans (0 was assigned if participant reported alcohol use) | Points MEPA score 100 15-16 80 12-14 50 8-11 25 4-7 0 0-3 |
| Physical Questionnaire  activity | | | - | Points Minutes 100 ≥420 90 360-419 80 300-359 60 240-299 40 120-239 20 1-119 0 0 |
| Nicotine Questionnaire  exposure | | | - | Points Status 100 Never tried 50 Tried but >30d ago 0 Current use within 30d Subtract 20 points (unless score is 0) for living with active indoor smoker in home |
| Sleep health | Questionnaire | | Recommendation from Healthy China Action (2019-2030) | Points Level 100 Age-appropriate optimal 90 <1h above optimal range 70 <1h below optimal range 40 1-<2h below or ≥1h above optimal 20 2-<3h below optimal range 0 ≥3h below optimal range |

| Table S2. Measurements and quantitative assessment criteria of Life's Essential 8 metrics (health factors). | | | |
| --- | --- | --- | --- |
| CVH metric | Method of measurement | Tools for evaluation | Quantification |
| Body mass index  Calculated as weight  (kg)/height^2^ (m^2^) | | 2007 Growth Reference from World Health Organization (age and sex specific) | Points Level 100 5th-<85th percentile 70 85th-<95th percentile 30 95th percentile-<120% of 95th percentile 15 <140% of 95th percentile 0 ≥140% of 95th percentile |
| Blood lipids | CHOD-PAP (Kyowa Medex Co., Ltd., Tokyo, Japan) method | - | Points Level 100 <100 70 100-119 30 120-144 15 145-189 0 ≥190 |
| Blood glucose GOD-PAP. (Randox Laboratories Ltd., London, UK) and high-performance liquid chromatography system (model HLC-723 G7; Tosoh Corporation, Tokyo, Japan) | | - | Points Level 100 No history of DM & FPG<100 (or HbA1c<5.7) 60 No DM & FPG 00-125 (or HbA1c 5.7-6.4) 40 DM with HbA1c<7.0 30 DM with HbA1c 7.0-7.9 20 DM with HbA1c 8.0-8.9 10 DM with HbA1c 9.0-9.9 0 DM with HbA1c ≥10 |
| Blood pressure With validated devices | | Clinical Practice Guideline for Screening and Management of High Blood Pressure in Children and Adolescents (2017) | Points Level 100 Optimal 75 Elevated 50 Stage 1 hypertension 25 Stage 2 hypertension 0 Systolic BP ≥160 or ≥95th percentile+30 mm Hg systolic BP, whichever is lower; and/ or diastolic BP ≥100 or ≥95th percentile+20 mm Hg diastolic BP |

| Table S3. Provinces (autonomous regions and municipalities) in each geographical region | | | | | | |
| --- | --- | --- | --- | --- | --- | --- |
| North China | Northeast China | East China | Central China | Southwest China | Northwest China | Hongkong/ Macau |
| Beijing | Heilongjiang | Shanghai | Henan | Chongqing | Shaanxi |  |
| Tianjin | Jilin | Jiangsu | Hubei | Sichuan | Gansu |  |
| Hebei | Liaoning | Zhejiang | Hunan | Guizhou | Qinghai |  |
| Shanxi | Neimenggu | Anhui | Guangdong | Yunnan | Ningxia |  |
|  |  | Fujian | Guangxi | Xizang | Xinjiang |  |
|  |  | Jiangxi | Hainan |  |  |  |
|  |  | Shandong |  |  |  |  |
|  |  | Taiwan |  |  |  |  |

| Table S4. Demographic characteristics of included and excluded participants of the present study. | | | | | | | |
| --- | --- | --- | --- | --- | --- | --- | --- |
| Characteristics | | Excluded | |  | Included | | *P* for difference |
|  |  | Sample size | Distribution |  | Sample size | Distribution |  |
| Total number |  | 6556 |  |  | 21921 |  |  |
| Urban, n (%) |  | 6556 | 1486 (22.7) |  | 21921 | 6071 (27.7) | 0.67 |
| Sex, male, n (%) |  | 6556 | 3431 (52.3) |  | 21921 | 11537 (52.6) | <.001 |
| Age, year, mean (SD) |  | 6556 | 14.2 (3.8) |  | 21921 | 13.0 (3.6) | <.001 |
| Diet score, mean (SD) |  | 873 | 31.6 (11.2) |  | 20285 | 28.4 (9.0) | <.001 |
| Physical activity, n (%) | ≥ 420 min | 3974 | 2469 (62.1) |  | 17767 | 5477 (30.8) | <.001 |
|  | 360-419 min |  | 22 (0.6) |  |  | 701 (4.0) |  |
|  | 300-359 min |  | 16 (0.4) |  |  | 940 (5.3) |  |
|  | 240-299 min |  | 15 (0.4) |  |  | 1041 (5.9) |  |
|  | 120-239 min |  | 39 (1.0) |  |  | 3259 (18.3) |  |
|  | 1-119 min |  | 46 (1.2) |  |  | 3300 (18.6) |  |
|  | Never |  | 1367 (34.4) |  |  | 3049 (17.2) |  |
| Cigarette use, n (%) | Never tried | 49 | 46 (93.9) |  | 10921 | 10356 (94.8) | 0.92 |
|  | Tried, but > 30 days ago | | - |  |  | 13 (0.1) |  |
|  | Use within 30 days | | 3 (6.1) |  |  | 552 (5.1) |  |
| Sleep per night, hours, mean (SD) |  | 3 | 10.7 (1.2) |  | 9831 | 8.7 (1.2) | 0.002 |
| Height, cm, mean (SD) |  | 1062 | 150.1 (22.7) |  | 19167 | 146.6 (16.8) | <.001 |
| Weight, kg, mean (SD) |  | 1099 | 48.1 (24.5) |  | 19167 | 40.1 (14.4) | <.001 |
| Body mass index, kg/m^2^, mean (SD) |  | 983 | 19.7 (4.8) |  | 19167 | 18.0 (3.2) | <.001 |
| Non-HDL-C, mg/dL, mean (SD) |  | NA |  |  |  |  |  |
| Fasting plasma glucose, mg/dL, mean (SD) |  | NA |  |  |  |  |  |
| Systolic blood pressure, mmHg, mean (SD) |  | 173 | 98.2 (14.9) |  | 19062 | 99.9 (13.5) | 0.10 |
| Diastolic blood pressure, mmHg, mean (SD) |  | 171 | 65.7 (10.3) |  | 19055 | 65.4 (9.8) | 0.73 |
| SD, standard deviation; NA, not applicable. |  |  |  |  |  |  |  |

| Table S5. Sample distribution of each research wave by geographical regions, 1989-2018. | | | | | | | | | |
| --- | --- | --- | --- | --- | --- | --- | --- | --- | --- |
| Wave | Sample size | Age (mean, SD) | Sex (male%) | Geographic distribution (%) | | | | | |
|  |  |  |  | North China | Northeast China | East China | Central China | Southwest China | Northwest China |
| 1989 | 237 | 13.4 (5.0) | 47.3 | - | 14.8 | 18.6 | 56.1 | 10.6 | - |
| 1991 | 2976 | 13.7 (3.7) | 51.2 | - | 9.3 | 20.1 | 51.8 | 18.8 | - |
| 1993 | 2665 | 13.4 (3.7) | 51.6 | - | 10.1 | 20.5 | 52.3 | 17.2 | - |
| 1997 | 3194 | 13.3 (3.7) | 53.4 | - | 10.0 | 20.4 | 56.1 | 13.6 | - |
| 2000 | 2788 | 13.4 (3.3) | 53.2 | - | 21.5 | 18.2 | 47.9 | 12.5 | - |
| 2004 | 1801 | 13.4 (3.4) | 53.6 | - | 21.9 | 15.8 | 49.6 | 12.8 | - |
| 2006 | 1448 | 13.1 (3.6) | 54.1 | - | 20.9 | 15.0 | 48.1 | 16.0 | - |
| 2009 | 1244 | 12.8 (3.5) | 55.6 | - | 15.4 | 17.8 | 51.3 | 15.5 | - |
| 2011 | 1584 | 12.4 (3.5) | 51.1 | 8.7 | 8.8 | 21.5 | 37.6 | 23.5 | - |
| 2015 | 2071 | 11.8 (3.3) | 52.4 | 6.3 | 5.9 | 22.2 | 28.7 | 25.6 | 11.3 |
| 2018 | 1913 | 11.7 (3.2) | 52.6 | 7.1 | 5.0 | 21.2 | 31.2 | 23.4 | 12.0 |

Figure S1. Temporal changes of cardiovascular health metrics in Chinese school aged children and adolescents from 1989 to 2018, and the differences across geographical regions.

A, Life’s Essential 8 score; B, Health behaviors score (total); C, Health factors score (total); D, Dietary; E, Physical activity; F, Nicotine exposure; G, Sleep health; H, Body mass index; I, non-HDL cholesterol; J, Fasting plasma glucose; K, Blood pressure.

Models were adjusted for age, sex and urban residence of each participant.
